# Supplementary material for: Assessing the strength of case growth trends in the coronavirus pandemic
Source: R Soc Open Sci. 2020 Nov 25;7(11):201622. doi: 10.1098/rsos.201622 (PMC7735351; doi:10.1098/rsos.201622)
Supplement: Supplementary Material [file rsos201622supp2.pdf]

Supplementary Material for

Assessing the strength of case growth trends in the coronavirus pandemic

Levente Kriston[1]

[1] Department of Medical Psychology, University Medical Center Hamburg-Eppendorf, Martinistr. 52, 20246 Hamburg, Germany

Correspondence should be addressed to:

Levente Kriston

Department of Medical Psychology

University Medical Center Hamburg-Eppendorf

Martinistr. 52

D-20246 Hamburg

Germany

E-mail: l.kriston@uke.de

Tel: +49 (0)40 7410 56849

Includes:

- Supplementary Figures S1 to S12
- Captions for Supplementary Data S1, Supplementary Data S2, and Supplementary Code S1

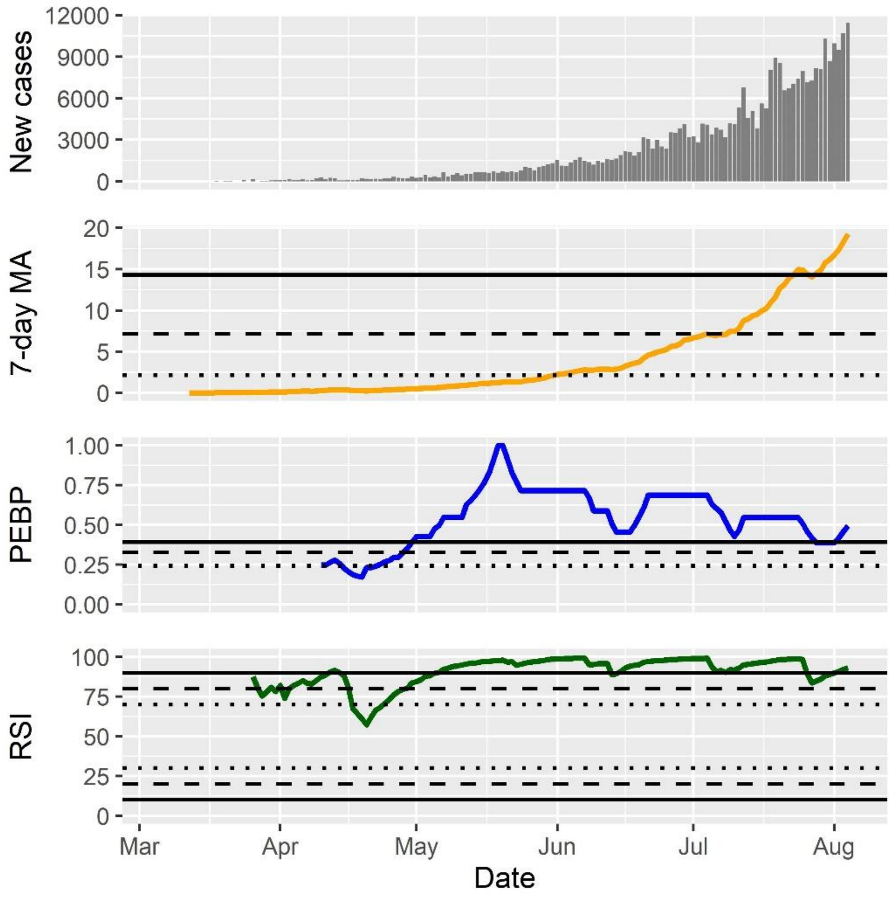

Fig. S1. Development of the investigated indicators in Colombia. For the 7-day moving average of the standardized incidence per 100,000 people (MA), dotted, dashed, and solid lines indicate thresholds for considerable, serious, and critical case counts, respectively. For the permutation entropy-based predictability (PEBP) and the relative strength index (RSI), dotted, dashed, and solid lines indicate thresholds for possible, likely, and highly probable trends, respectively.

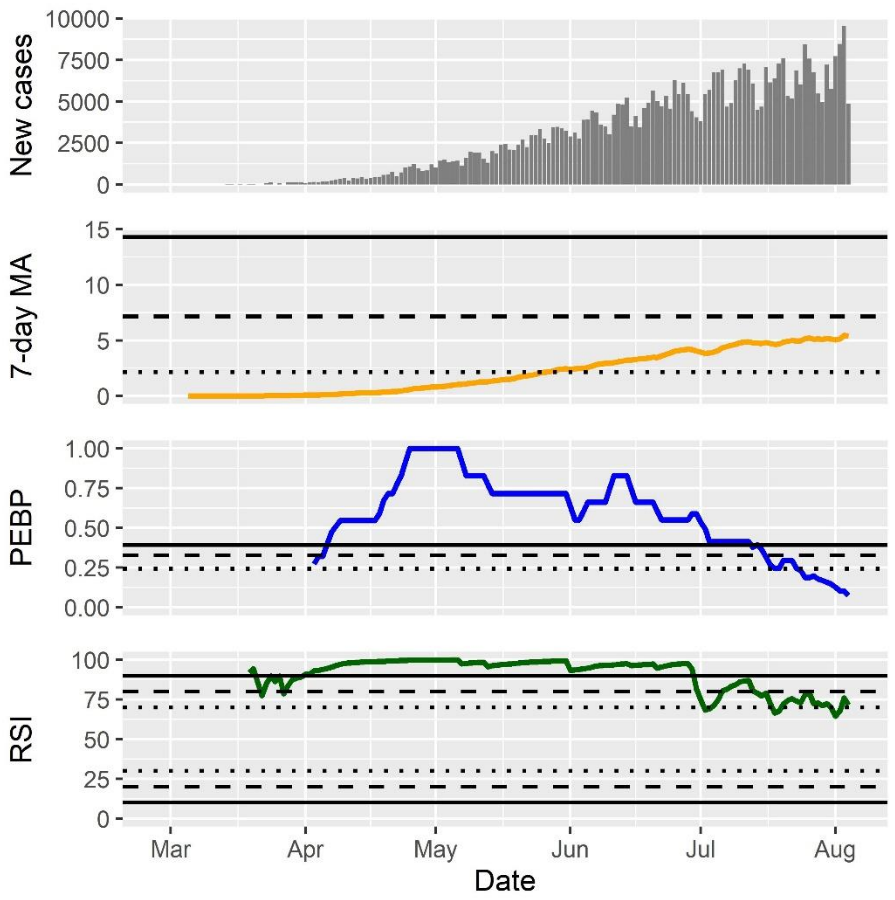

Fig. S2. Development of the investigated indicators in Mexico. For the 7-day moving average of the standardized incidence per 100,000 people (MA), dotted, dashed, and solid lines indicate thresholds for considerable, serious, and critical case counts, respectively. For the permutation entropy-based predictability (PEBP) and the relative strength index (RSI), dotted, dashed, and solid lines indicate thresholds for possible, likely, and highly probable trends, respectively.

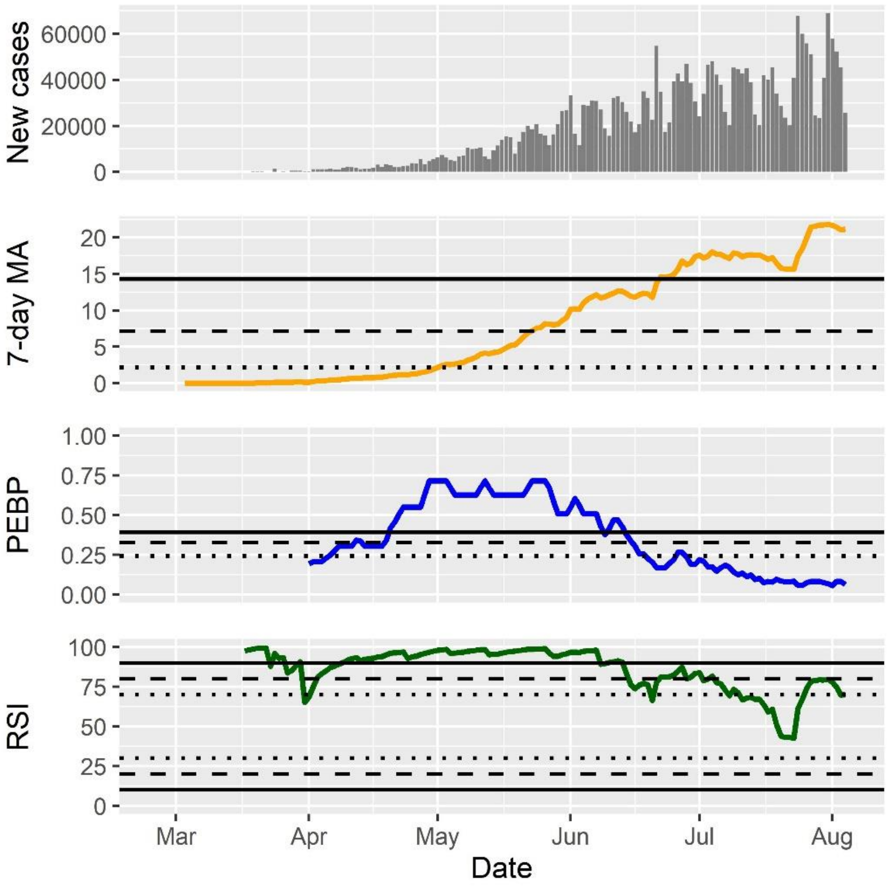

Fig. S3. Development of the investigated indicators in Brazil. For the 7-day moving average of the standardized incidence per 100,000 people (MA), dotted, dashed, and solid lines indicate thresholds for considerable, serious, and critical case counts, respectively. For the permutation entropy-based predictability (PEBP) and the relative strength index (RSI), dotted, dashed, and solid lines indicate thresholds for possible, likely, and highly probable trends, respectively.

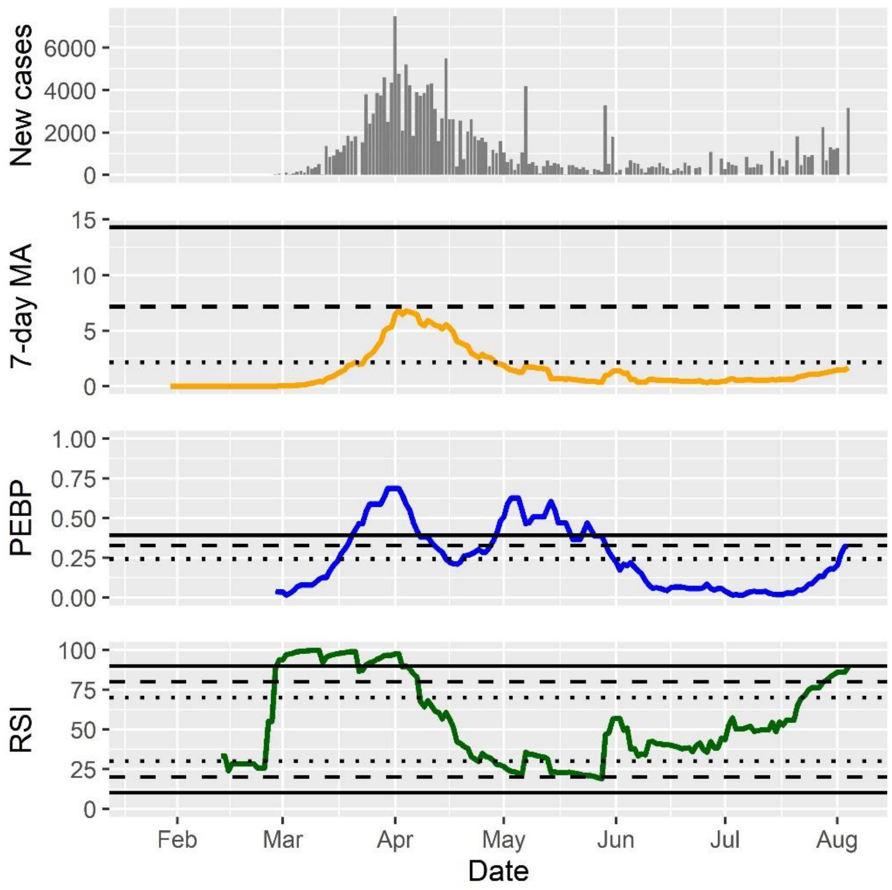

Fig. S4. Development of the investigated indicators in France. For the 7-day moving average of the standardized incidence per 100,000 people (MA), dotted, dashed, and solid lines indicate thresholds for considerable, serious, and critical case counts, respectively. For the permutation entropy-based predictability (PEBP) and the relative strength index (RSI), dotted, dashed, and solid lines indicate thresholds for possible, likely, and highly probable trends, respectively.

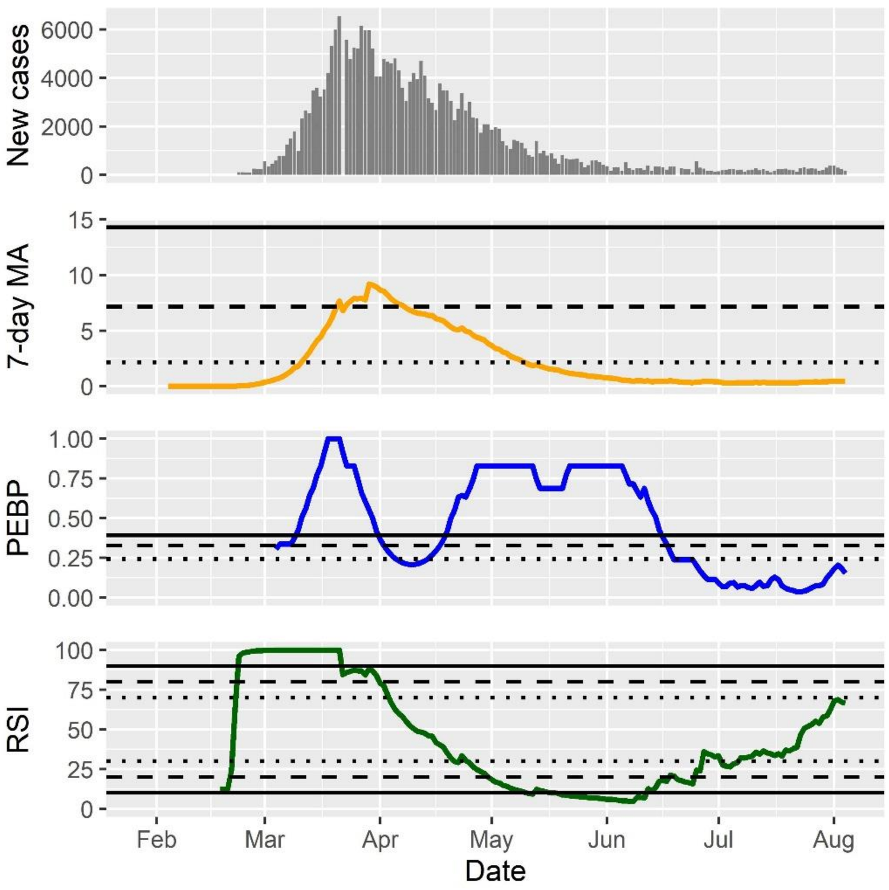

Fig. S5. Development of the investigated indicators in Italy. For the 7-day moving average of the standardized incidence per 100,000 people (MA), dotted, dashed, and solid lines indicate thresholds for considerable, serious, and critical case counts, respectively. For the permutation entropy-based predictability (PEBP) and the relative strength index (RSI), dotted, dashed, and solid lines indicate thresholds for possible, likely, and highly probable trends, respectively.

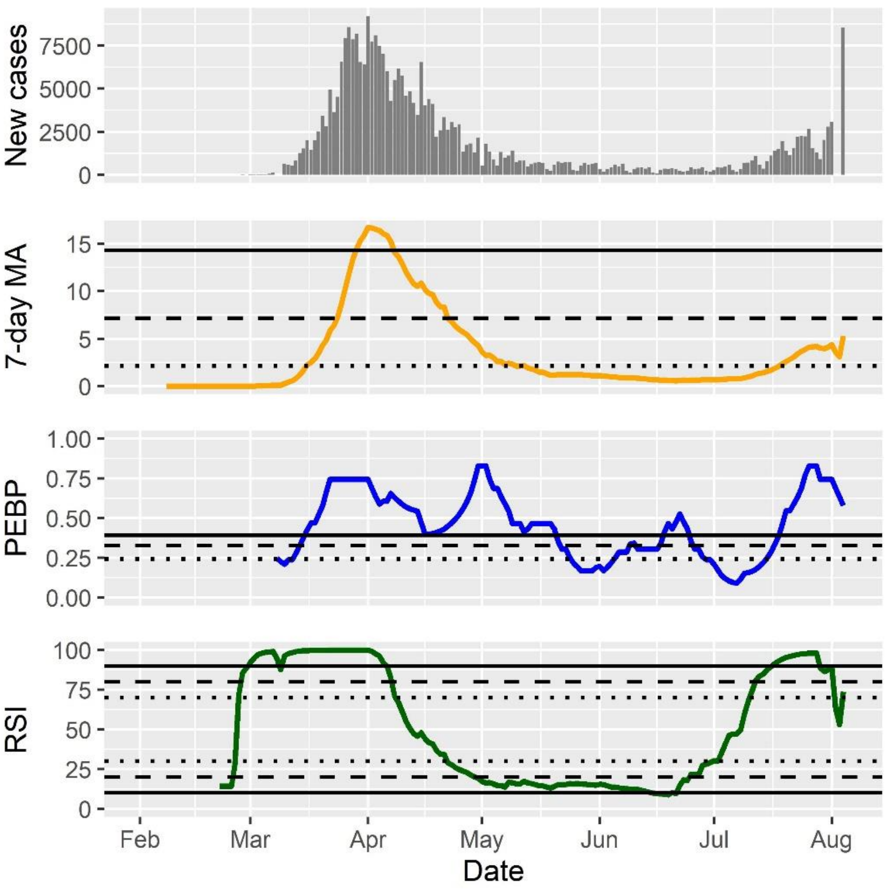

Fig. S6. Development of the investigated indicators in Spain. For the 7-day moving average of the standardized incidence per 100,000 people (MA), dotted, dashed, and solid lines indicate thresholds for considerable, serious, and critical case counts, respectively. For the permutation entropy-based predictability (PEBP) and the relative strength index (RSI), dotted, dashed, and solid lines indicate thresholds for possible, likely, and highly probable trends, respectively.

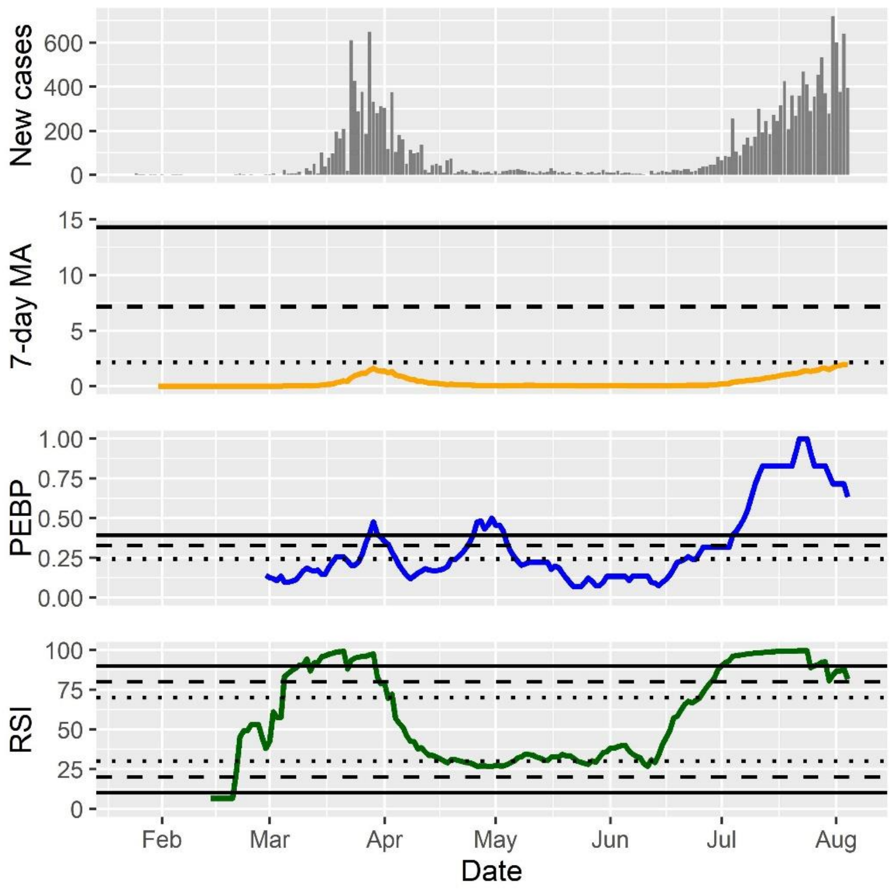

Fig. S7. Development of the investigated indicators in Australia. For the 7-day moving average of the standardized incidence per 100,000 people (MA), dotted, dashed, and solid lines indicate thresholds for considerable, serious, and critical case counts, respectively. For the permutation entropy-based predictability (PEBP) and the relative strength index (RSI), dotted, dashed, and solid lines indicate thresholds for possible, likely, and highly probable trends, respectively.

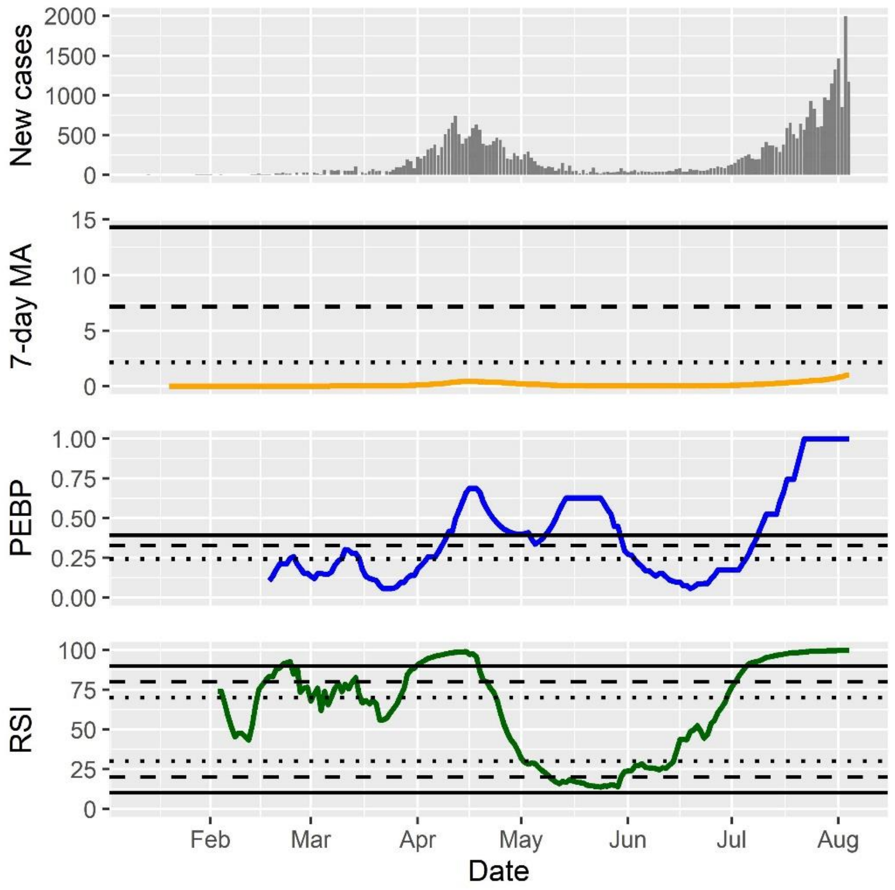

Fig. S8. Development of the investigated indicators in Japan. For the 7-day moving average of the standardized incidence per 100,000 people (MA), dotted, dashed, and solid lines indicate thresholds for considerable, serious, and critical case counts, respectively. For the permutation entropy-based predictability (PEBP) and the relative strength index (RSI), dotted, dashed, and solid lines indicate thresholds for possible, likely, and highly probable trends, respectively.

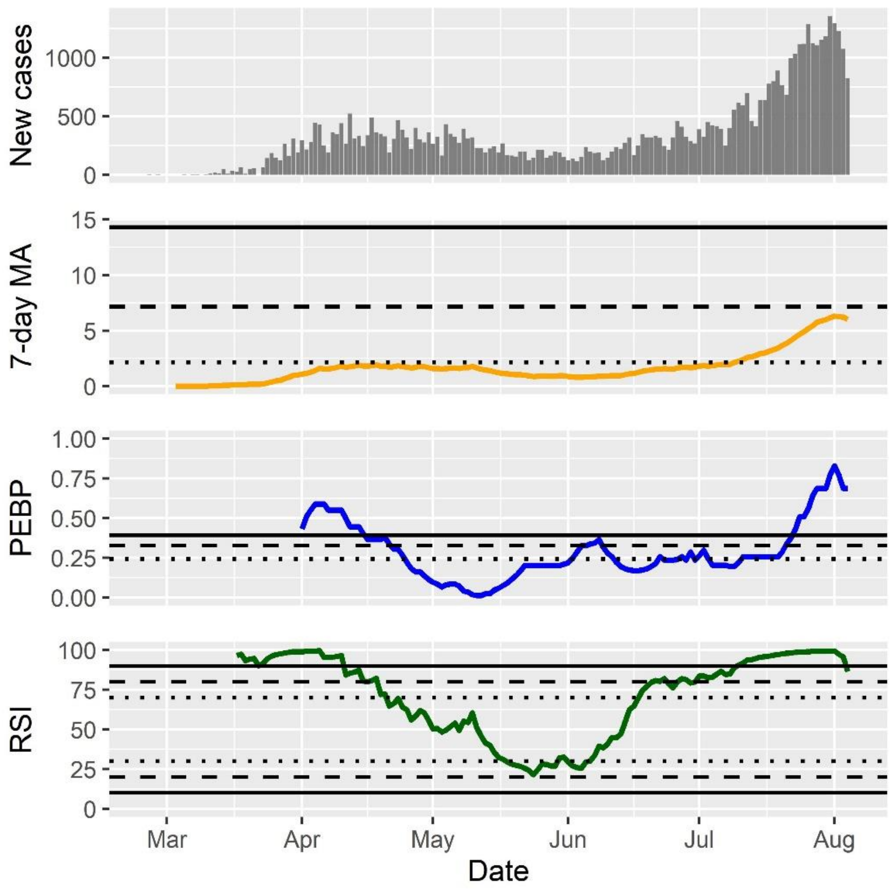

Fig. S9. Development of the investigated indicators in Romania. For the 7-day moving average of the standardized incidence per 100,000 people (MA), dotted, dashed, and solid lines indicate thresholds for considerable, serious, and critical case counts, respectively. For the permutation entropy-based predictability (PEBP) and the relative strength index (RSI), dotted, dashed, and solid lines indicate thresholds for possible, likely, and highly probable trends, respectively.

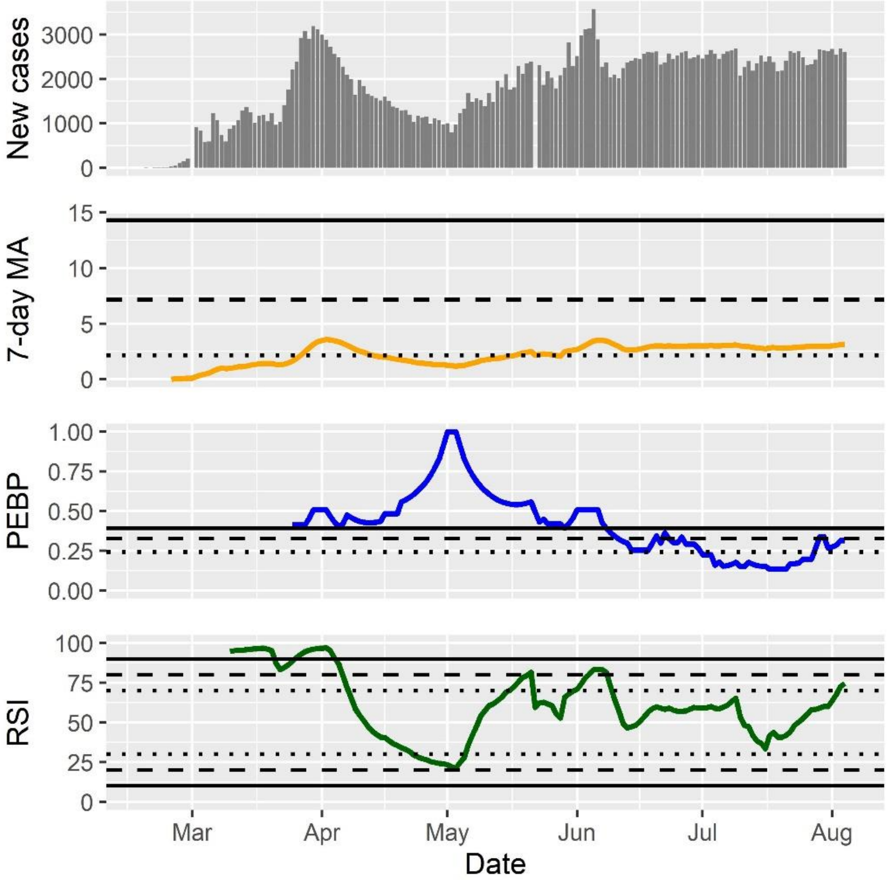

Fig. S10. Development of the investigated indicators in Iran. For the 7-day moving average of the standardized incidence per 100,000 people (MA), dotted, dashed, and solid lines indicate thresholds for considerable, serious, and critical case counts, respectively. For the permutation entropy-based predictability (PEBP) and the relative strength index (RSI), dotted, dashed, and solid lines indicate thresholds for possible, likely, and highly probable trends, respectively.

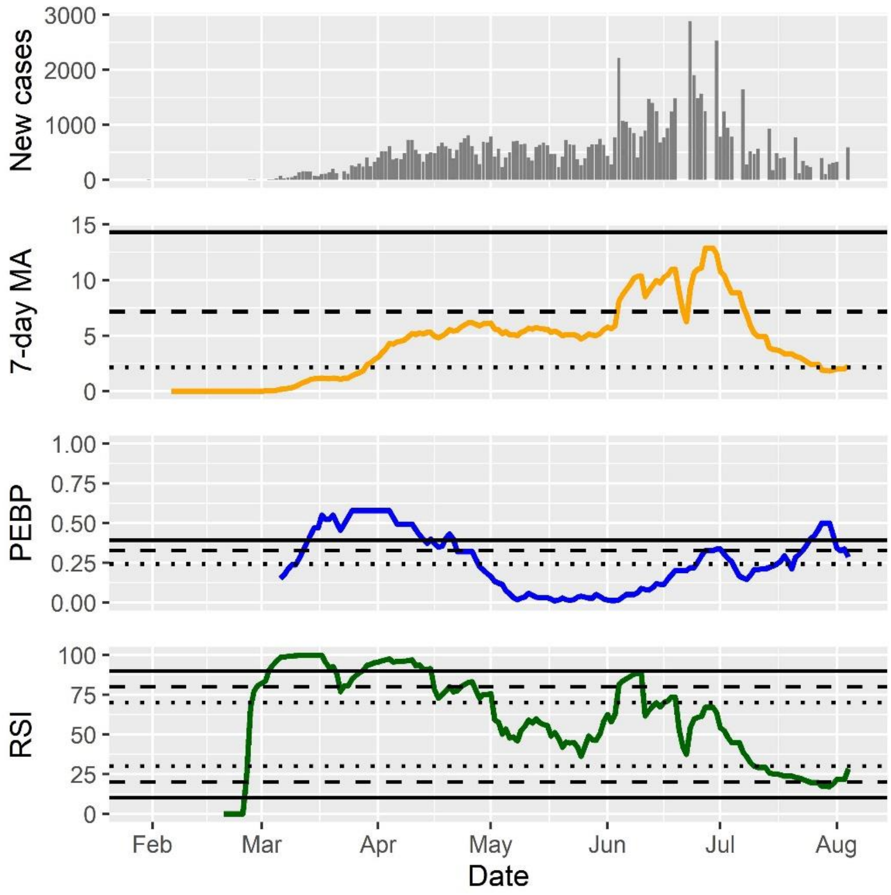

Fig. S11. Development of the investigated indicators in Sweden. For the 7-day moving average of the standardized incidence per 100,000 people (MA), dotted, dashed, and solid lines indicate thresholds for considerable, serious, and critical case counts, respectively. For the permutation entropy-based predictability (PEBP) and the relative strength index (RSI), dotted, dashed, and solid lines indicate thresholds for possible, likely, and highly probable trends, respectively.

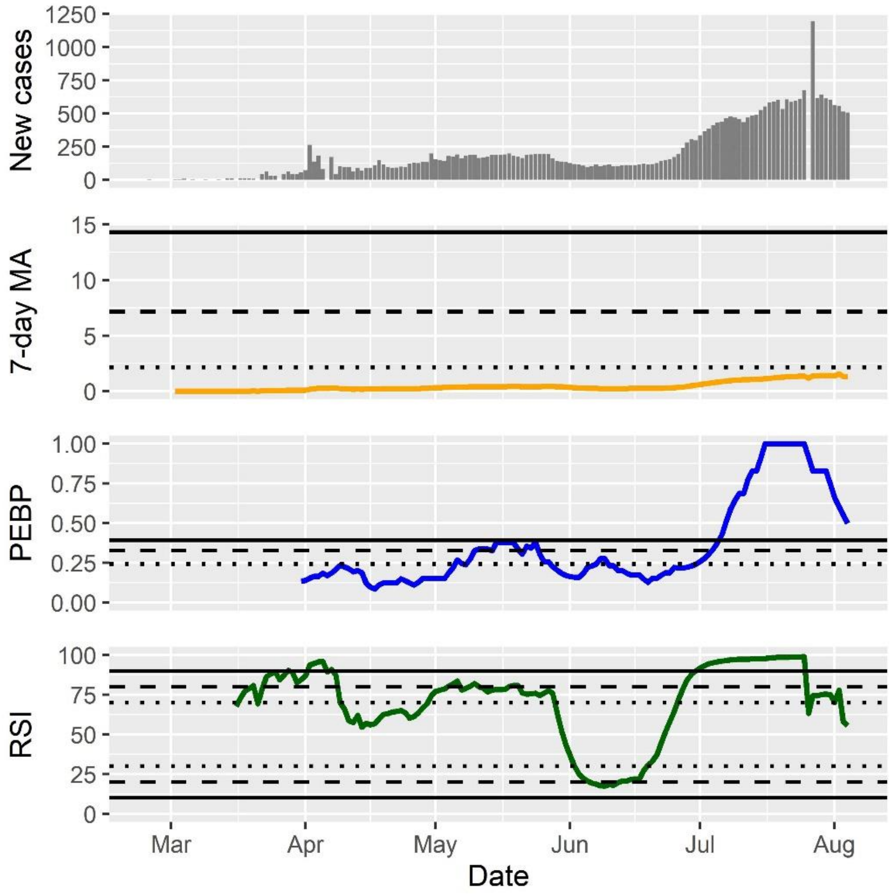

Fig. S12. Development of the investigated indicators in Algeria. For the 7-day moving average of the standardized incidence per 100,000 people (MA), dotted, dashed, and solid lines indicate thresholds for considerable, serious, and critical case counts, respectively. For the permutation entropy-based predictability (PEBP) and the relative strength index (RSI), dotted, dashed, and solid lines indicate thresholds for possible, likely, and highly probable trends, respectively.

Further supplements

Data S1. (separate file). Daily new case count data on 4 August 2020 (csv)

Data S2. (separate file). Country population data (csv)

Code S1. (separate file). R-code used for analysis (pdf)
