# Supplementary material for: Assessing the strength of case growth trends in the coronavirus pandemic
Source: R Soc Open Sci. 2020 Nov 25;7(11):201622. doi: 10.1098/rsos.201622 (PMC7735351; doi:10.1098/rsos.201622)
Supplement: Code S1 [file rsos201622supp4.pdf]

Assessing the strength of case growth trends in the coronavirus pandemic

Levente Kriston

5 August 2020

R code used for analysis

```
############# START ###############

### LOAD LIBRARIES

library(ggplot2)
library(statcomp)
library(grid)
library(caTools)
library(TTR)

### OPTIONS
options(max.print=1000000)
Sys.setlocale("LC_TIME", "English")

### DATA IMPORT

## Case count data imported directly from https://covid19.who.int
data <- read.csv("https://covid19.who.int/WHO-COVID-19-global-data.csv",
                 fileEncoding = "UTF-8-BOM",
                 stringsAsFactors=F)

## Alternatively, you can use the data attached to the manuscript
# Specify path or save data to working directory

# data <- read.csv("Cases_data_04-August-2020.csv", stringsAsFactors=F)

# Reformat datum
data$Date_reported <- as.Date(substr(data$Date_reported, 1, 10))

## Load population data attached to the manuscript
# Specify path or save data to working directory

data.pop <- read.csv("Country_pop_data.csv", stringsAsFactors=F)

### DEFINE COUNTRY FOR ANALYSIS VIA TWO-LETTER CODE
cid <- "IN"
```

```r
## Subset Case count data
data.sub <- data[which(data$Country_code==cid), ]

### CALCULATE INDICATORS AND CREATE PLOTS

## Daily new cases

# Plot daily new cases
plot.newcases.sub <- ggplot(data.sub, aes(Date_reported, New_cases)) +
  geom_col(width=.9, fill="gray50") +
  scale_x_date(limits=c(head(data.sub$Date_reported, 1)-1, tail(data.sub$Date_reported, 1)+1),
               breaks="month") +
  scale_y_continuous(name="New cases",
                     limits=c(0, NA)) +
  theme(
    axis.title.x = element_blank(),
    axis.text.x = element_blank(),
    axis.ticks.x=element_blank())

## Moving average (MA)

# Calculate raw MA
data.sub$ma <- runmean(data.sub$New_cases, 7, endrule="NA", align="right")

# Calculate standardized MA (uses Population data)
data.sub$ma.st <- data.sub$ma/data.pop[which(data.pop$cca2==cid), "pop2020"]*100

# Plot MA
plot.ma.sub <- ggplot(data.sub, aes(Date_reported, ma.st)) +
  geom_line(col="orange", size=1) +
  geom_hline(yintercept = 2.14, size=.7, linetype=3) +
  geom_hline(yintercept = 7.14 , size=.7, linetype=2) +
  geom_hline(yintercept = 14.29, size=.7, linetype=1) +
  scale_x_date(limits=c(head(data.sub$Date_reported, 1)-1, tail(data.sub$Date_reported, 1)+1),
               breaks="month") +
  scale_y_continuous(name="7-day MA",
                     limits=c(0, NA)) +
  theme(
    axis.title.x = element_blank(),
    axis.text.x = element_blank(),
    axis.ticks.x=element_blank())

## Permutation entropy-based prediction (PEBP)

# Calculate PEBP
set.seed(20200622)

ts.wnoise.ma <- data.sub$ma + rnorm(nrow(data.sub), 0, .01) # Add random pertubations to data

pe.30.ma <- numeric() # initialize vector for permutation entropy
for (i in 36:(length(ts.wnoise.ma))){
  pe.30.ma[i] <- permutation_entropy(ordinal_pattern_distribution(ts.wnoise.ma[(i-29):i], 3))
}
```

```
data.sub$pred.30.ma <- 1-pe.30.ma # calculate prediction from entropy

# Define thresholds for PEBP through simulation
# not needed for figures
# only for reasons of replicability
set.seed(007008)
nrep <- 100000 # number of simulations
m <- matrix(0, nrep, 36) # initialize matrix
pred <- numeric(nrep) # initalize output vector
for (i in 1:nrep){
  m[i, ] <- rnorm(36, 0, 1)
  pred[i] <- 1-permutation_entropy(ordinal_pattern_distribution(
    runmean(m[i ,], 7, endrule="NA", align="right"),
    3))
}
quantile(pred, probs=c(.99, .999, .9999)) # Calculate quantiles

# Plot PEBP
plot.pe.sub <- ggplot(data.sub, aes(Date_reported, pred.30.ma)) +
  geom_line(col="blue", size=1, linetype=1) +
  geom_hline(yintercept = .242, size=.7, linetype=3) +
  geom_hline(yintercept = .326, size=.7, linetype=2) +
  geom_hline(yintercept = .391, size=.7, linetype=1) +
  scale_x_date(limits=c(head(data.sub$Date_reported, 1)-1, tail(data.sub$Date_reported, 1)+1),
               breaks="month") +
  scale_y_continuous(name="PEBP",
                     limits=c(0, 1)) +
  theme(
    axis.title.x = element_blank(),
    axis.text.x = element_blank(),
    axis.ticks.x=element_blank())

## Relative strentgh index (RSI)

# Calculate RSI
data.sub$rsi <- RSI(data.sub$ma, n=14)

# Plot RSI
plot.rsi.sub <- ggplot(data.sub, aes(Date_reported, rsi)) +
  geom_line(col="darkgreen", size=1, linetype=1) +
  geom_hline(yintercept = c(30,70), size=.7, linetype=3) +
  geom_hline(yintercept = c(20,80), size=.7, linetype=2) +
  geom_hline(yintercept = c(10,90), size=.7, linetype=1) +
  scale_x_date(name="Date",
    limits=c(head(data.sub$Date_reported, 1)-1, tail(data.sub$Date_reported, 1)+1),
               breaks="month",
    date_labels = "%b") +
  scale_y_continuous(name="RSI",
                     limits=c(0, 100))

## Create combined plot
grid.newpage()
grid.draw(rbind(ggplotGrob(plot.newcases.sub),
```

```
                 ggplotGrob(plot.ma.sub),
                 ggplotGrob(plot.pe.sub),
                 ggplotGrob(plot.rsi.sub),
                 size = "first"))

############# END ###############
```
